# Squamous Cell Carcinoma of the Anus Incidence, Mortality, and Survival Among the General Population and Persons Living With HIV in Puerto Rico, 2000-2016

**DOI:** 10.1200/GO.20.00299

**Published:** 2021-01-25

**Authors:** Karen J. Ortiz-Ortiz, Jeslie M. Ramos-Cartagena, Ashish A. Deshmukh, Carlos R. Torres-Cintrón, Vivian Colón-López, Ana P. Ortiz

**Affiliations:** ^1^ Division of Cancer Control and Population Sciences, University of Puerto Rico Comprehensive Cancer Center, San Juan, Puerto Rico; ^2^ Department of Health Services Administration, Graduate School of Public Health, Medical Sciences Campus, University of Puerto Rico, San Juan, Puerto Rico; ^3^ Puerto Rico Central Cancer Registry, University of Puerto Rico Comprehensive Cancer Center, San Juan, Puerto Rico; ^4^ University of Puerto Rico/MD Anderson Cancer Center Partnership for Excellence in Cancer Research Program, San Juan, Puerto Rico; ^5^ Center for Health Services Research, Department of Management, Policy, and Community Health, UTHealth School of Public Health, Houston, TX; ^6^ Department of Biostatistics and Epidemiology, Graduate School of Public Health, Medical Sciences Campus, University of Puerto Rico, San Juan, Puerto Rico

## Abstract

**PURPOSE:**

Squamous cell carcinoma of the anus (SCCA) is common among persons living with HIV (PLWH). We described SCCA incidence and survival among the general population and among PLWH in Puerto Rico (PR), along with mortality of anal cancer.

**METHODS:**

PR HIV/AIDS Surveillance Program and the PR Central Cancer Registry databases were linked (2000-2016). Incidence rates (IRs) and trends (annual percent change [APC]) in SCCA and mortality rates and trends for anal cancer were estimated. Relative survival and relative excess risk (RER) of death were calculated.

**RESULTS:**

From 2000 to 2016, 991 individuals in PR were diagnosed with anal cancer; 73% of cases were SCCA 9.1% of SCCA and 1.5% of non-SCCA cases were in PLWH (*P* < .0001). SCCA incidence was higher among PLWH than the general population (IR = 27.7/100,000). Among PLWH, SCCA incidence (per 100,000) was the highest among men who have sex with men (IR = 60.5). From 2001-2016, SCCA incidence increased among the general population (APC: 4.90, *P* < .05); however, no significant change was observed among PLWH (APC = 0.19 and *P* = .96). The APC for anal cancer mortality in the general population was positive (3.9%) from 2000 to 2016, but not significant (*P* > .05). The 5-year relative survival of SCCA was 56.9% among PLWH and 66.8% among the general population. In multivariate analysis, the RER of death for SCCA 5 years postdiagnosis was affected by stage at diagnosis (distant: RER = 7.6, 95% CI, 2.36 to 24.25) but not by PLWH status (RER = 1.4, 95% CI, 0.67 to 3.01).

**CONCLUSION:**

Our findings highlight the relevance of anal cancer screening in PLWH and HPV vaccination in both PLWH and the general population in PR, which could have an impact on the disease trend in the next few decades.

## INTRODUCTION

Squamous cell carcinoma of the anus (SCCA) is the most common form of anal cancer caused by human papillomavirus infection (HPV). SCCA represents nearly 85%-90% of all anal cancer cases, whereas a minority are adenocarcinomas, a histology that is rarer but more aggressive than SCCA.^1–4^ Around 80%-90% of SCCA cases are caused by a high-risk infection with HPV.^3^ A recent publication that described incidence trends of anal cancer in the general population of the United States showed that the SCCA incidence significantly increased 2.7% annually during 2001-2015.^5^ As for persons living with HIV/AIDS (PLWH) in the United States, from 1996 to 2000, there was a 32.8% annual increase (*P* < .05) in the incidence of anal cancer, and then trends appeared to have stabilized from 2001 to 2008 (an annual increase of 1.4%), followed by a nonstatistically significant decline from 2008 to 2012 (an annual increase of −7.2%).^6^

**CONTEXT**

**Key Objective**
This study assesses the incidence, mortality, and survival of anal cancer among persons living with HIV (PLWH) and the general population living in Puerto Rico (PR).
**Knowledge Generated**
Our findings indicate that the rates of squamous cell carcinoma of the anus (SCCA) have increased dramatically among the general population living in PR. Although the incidence rate remains substantially greater among PLWH, the rise in incidence seems to have moderated in this risk group. Meanwhile, the relative excess risk of death for SCCA 5 years postdiagnosis was affected by stage at diagnosis but not by HIV status.
**Relevance**
Our study results support the need to determine optimal anal cancer screening strategies in PLWH in PR, whereas continuous improvement in human papillomavirus infection vaccine uptake is also needed, to have an impact on SCCA trends over the next decades.

It has been established that certain groups (ie, PLWH, men who have sex with men [MSM], and women with a history of lower genital dysplasia) are at a disproportionately elevated risk of SCCA.^7–10^ Studies indicate that PLWH in the United States have a 19-fold excess risk of anal cancer than the general population.^6,11^ The NCI HIV/AIDS cancer match study has also reported that among PLWH, the most affected group was MSM, having a 38-fold higher risk of anal cancer.^6^ Another study in the United States found that anal cancer was the most common HPV-related cancer among PLWH.^12^

There are around 1.1 million PLWH in the United States.^13^ A decrease in HIV-related deaths has been documented since the introduction of antiretroviral therapy (ART), which led to higher life expectancy contributing to increased risk of the development of certain cancers in this population.^14,15^ In particular, ART has caused a decrease in AIDS-defining cancers and an increase in non–AIDS-defining cancers, such as anal cancer.^11^ Puerto Rico (PR) ranks 12th in HIV prevalence among all states and territories in the United States^16^; there were 20,160 PLWH in 2016.^17^ From 1985 to 2005, anal cancer represented the HPV-related malignancy with the highest excess risk among PLWH in PR as compared to the general population.^18^ Nonetheless, trends in SCCA incidence and mortality rates, as well as survival in PR, are still unknown. Given the rising burden of HIV in PR, it is imperative to study the epidemiology of anal cancer in PLWH in this population to inform future optimal prevention efforts. Our objective was to describe incidence, mortality, and survival of SCCA among PLWH and the general population living in PR during 2000-2016.

## METHODS

### Data Sources

We used the PR Central Cancer Registry (PRCCR) and the PR HIV/AIDS Surveillance Program databases. The PR HIV/AIDS Surveillance Program collects demographic information and clinical characteristics of persons with HIV/AIDS living in PR. The PRCCR collects demographic characteristics and clinical information of all cancer cases diagnosed or treated in PR; it is recognized as a Registry of Excellence by the National Program of Cancer Registries and has a Gold certification by the North American Association of Central Cancer Registries (NAACCR). Both the HIV/AIDS Surveillance Program and the PRCCR linked their databases with the mortality files from the PR Department of Health’s Statistical Analysis Division to obtain information on vital status and cause of death.

### Linkage Process

The PR HIV/AIDS Surveillance Program and the PRCCR have had a collaboration since 2012 to link both databases periodically. The last linkage was performed for data from 1987 to 2016. To perform this match, we used Match*Pro v1.6, a probabilistic record linkage program developed by IMS (Calverton, Maryland), under contract for the NCI and supported by SEER.

### Study Population

Analysis included invasive anal cancer patient cases diagnosed in PR between 2000 and 2016, coded according to the International Classification of Diseases for Oncology 3rd (ICD-O-3) site codes C21.0-21.8, excluding histological codes 9050-9055 and 9590-9992. We used histological codes 8050-8076, 8083-8084, and 8123-8124 to identify SCCA cases. Only microscopically confirmed cases were included. Cases with unknown age and unknown sex and those identified only by death certificate or at autopsy were excluded from the analysis (Data Supplement). Deaths due to anal cancer were identified through the International Classification of Diseases, 10th Edition (ICD-10) using code C21.

### Study Variables

Demographic characteristics assessed included sex (male and female) and age at cancer diagnosis (< 45, 45-59, and ≥ 60 years). Among clinical characteristics, anal cancer cases were categorized as SCCA or non-SCCA. The period of SCCA diagnosis was categorized into years 2000-2005, 2006-2011, and 2012-2016; and stage at anal cancer diagnosis was defined as localized, regional, distant, and unknown. HIV status (yes or no), mode of HIV transmission (MSM, injection drug users [IDUs], MSM/IDU, heterosexual male, heterosexual female, and others), and AIDS diagnosis were obtained from the HIV/AIDS Surveillance System.

### Statistical Analysis

To compare the sociodemographic characteristics of the study population by cancer and HIV status, we used χ^2^ tests. *P* values were corrected using the Bonferroni-Holm method for multiple comparisons.^19^ Anal cancer incidence and mortality rates were expressed as the number of cases per 100,000 person-years. Longitudinal trends in the incidence of SCCA in PR were also evaluated for the 2001-2016 period; the year 2000 was eliminated from this analysis as a result of a lack of cases among women with HIV. Incidence trends were summarized using the annual percent change (APC) and were presented for overall cases, by sex and by age group (< 45, 45-59, and ≥ 60 years). Mortality trends were also calculated for anal cancer. The APCs were calculated using the Joinpoint Regression Program (version 4.8.0.1).^20^

One-, 3-, and 5-year relative survival rates were calculated for PLWH and the general population for the 2000-2012 period. The relative survival rate represents the ratio of the observed survival of patients with cancer divided by the expected survival for a group of people in a general population that is similar to that of the patient group with respect to race, sex, age, and calendar period of observation.^21^ General population refers to all individuals, including those with anal cancer diagnosis. Relative survival rates were estimated using the expected survival (Ederer II method) and based on the decennial life table for the population of PR, considering the population distribution by age, sex, and calendar year. We used the Poisson regression model to estimate the relative excess risk (RER) of death between variables of interest. The likelihood ratio test statistics were used to assess the significance of interaction terms. Stata/SE version 15.2 statistical software (Stata, College Station, TX) was used to perform the analyses. This study was approved by the Institutional Review Board of the University of PR Comprehensive Cancer Center.

## RESULTS

During 2000-2016, anal cancer was diagnosed in 991 individuals in PR. Of those individuals, 73% cases were SCCA and 64.5% were women (Table 1). The median age at diagnosis was 65 years for SCCA and 66 years for non-SCCA; more than two-thirds of cases in both groups were among individuals age 60 years and older. Analysis stratified by cancer histology showed that a greater proportion of SCCA cases were diagnosed among women (72.7%), whereas non-SCCA cases were more frequent among men (57.6%). Nine percent of SCCA cases were among PLWH as compared to only 1.5% of non-SCCA cases (*P* < .001) (Table 1). Most of the SCCA (55.0%) and non-SCCA cases (45.4%) were diagnosed at a localized stage. When cases of SCCA were compared by HIV status, SCCA among PLWH was more frequent in men (83.3%), whereas in HIV-negative individuals, it was more frequent in women (78.4%) (Table 2). Most of the SCCA cases in PLWH were observed in individuals age 45-59 years (48.5%) (30.3% in age < 45 years and 21.2% in age 60 years and older), whereas a large proportion of HIV-negative SCCA cases (70.7%) were observed among individuals age 60 years and older (*P* < .001). No significant differences were seen in the period of SCCA diagnosis (*P* = .257) and stage at diagnosis (*P* = .972).

**TABLE 1:**
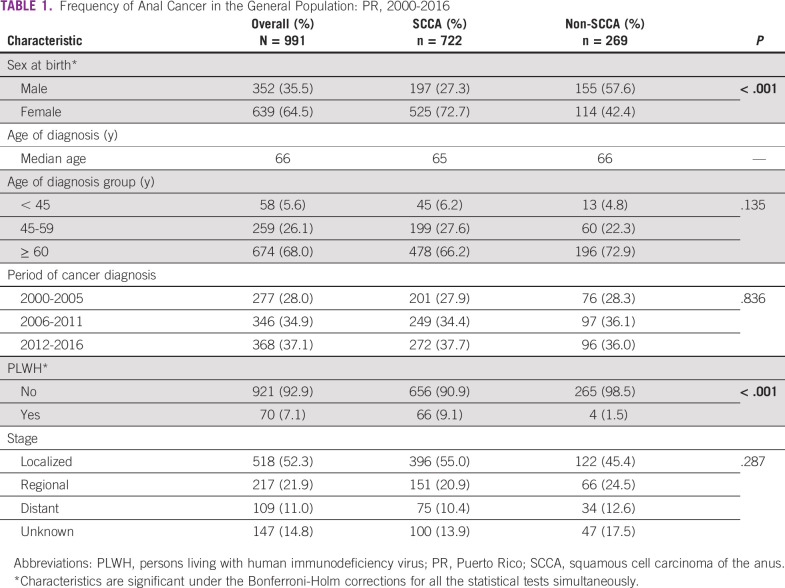
Frequency of Anal Cancer in the General Population: PR, 2000-2016

Abbreviations: PLWH, persons living with human immunodeficiency virus; PR, Puerto Rico; SCCA, squamous cell carcinoma of the anus.

* Characteristics are significant under the Bonferroni-Holm corrections for all the statistical tests simultaneously.

**TABLE 2:**
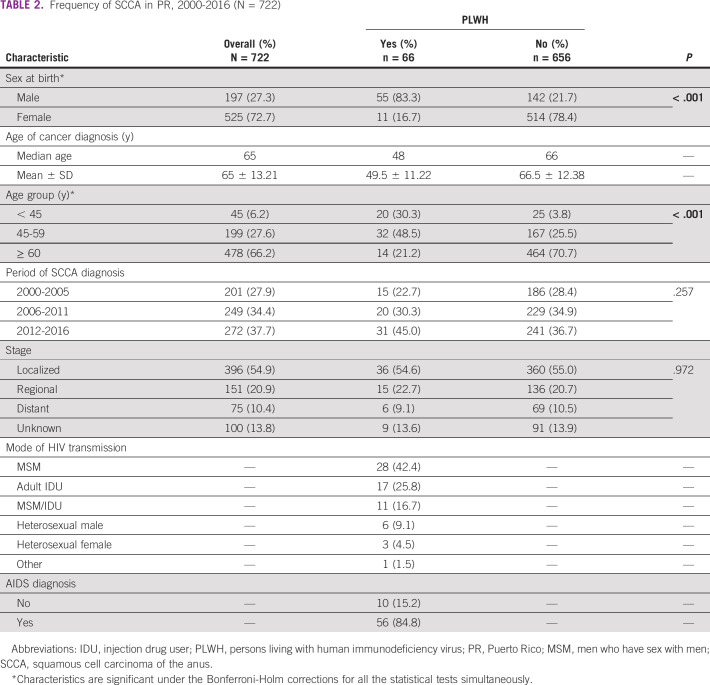
Frequency of SCCA in PR, 2000-2016 (N = 722)

Abbreviations: IDU, injection drug user; PLWH, persons living with human immunodeficiency virus; PR, Puerto Rico; MSM, men who have sex with men; SCCA, squamous cell carcinoma of the anus.

* Characteristics are significant under the Bonferroni-Holm corrections for all the statistical tests simultaneously.

Overall, the incidence of SCCA among PLWH was 27.7 per 100,000 person-years, whereas among the general population, it was 1.1 per 100,000 person-years (Table 3). The IR among men living with HIV was 33.6 per 100,000, whereas among women living with HIV, it was 14.7 per 100,000. Among PLWH, MSM who were IDUs had the highest incidence (77.1 per 100,000) followed by MSM (60.5 per 1000,000). PLWH with previous AIDS diagnosis had an incidence rate of 32.5 per 100,000, whereas individuals without a previous AIDS diagnosis had an incidence rate of 15.1 per 100,000. For the general population and PLWH, incidence rates of SCCA increased by age groups, which are lowest among individuals younger than 45 years (PLWH, 19.3 per 100,000; general population, 0.1 per 100,000) and highest among individuals 60 years and older (PLWH, 52.3 per 100,000; general population, 3.9 per 100,000) (Table 3).

**TABLE 3:**
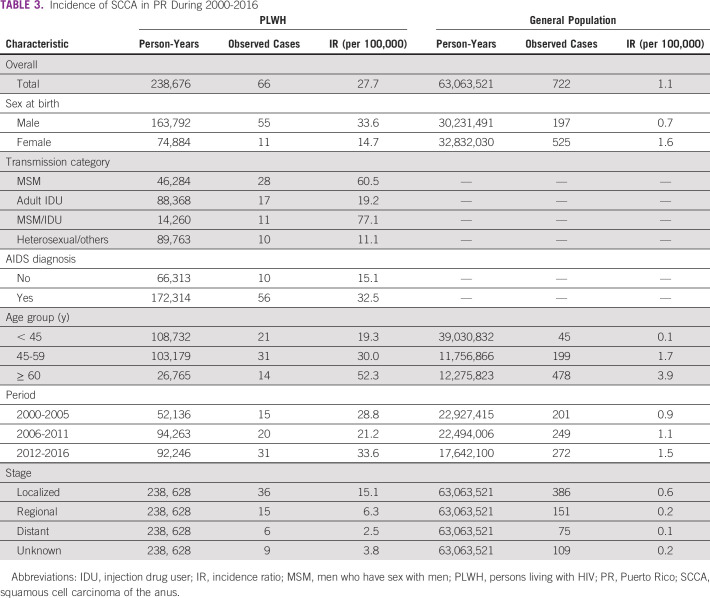
Incidence of SCCA in PR During 2000-2016

Abbreviations: IDU, injection drug user; IR, incidence ratio; MSM, men who have sex with men; PLWH, persons living with HIV; PR, Puerto Rico; SCCA, squamous cell carcinoma of the anus.

**FIG 1:**
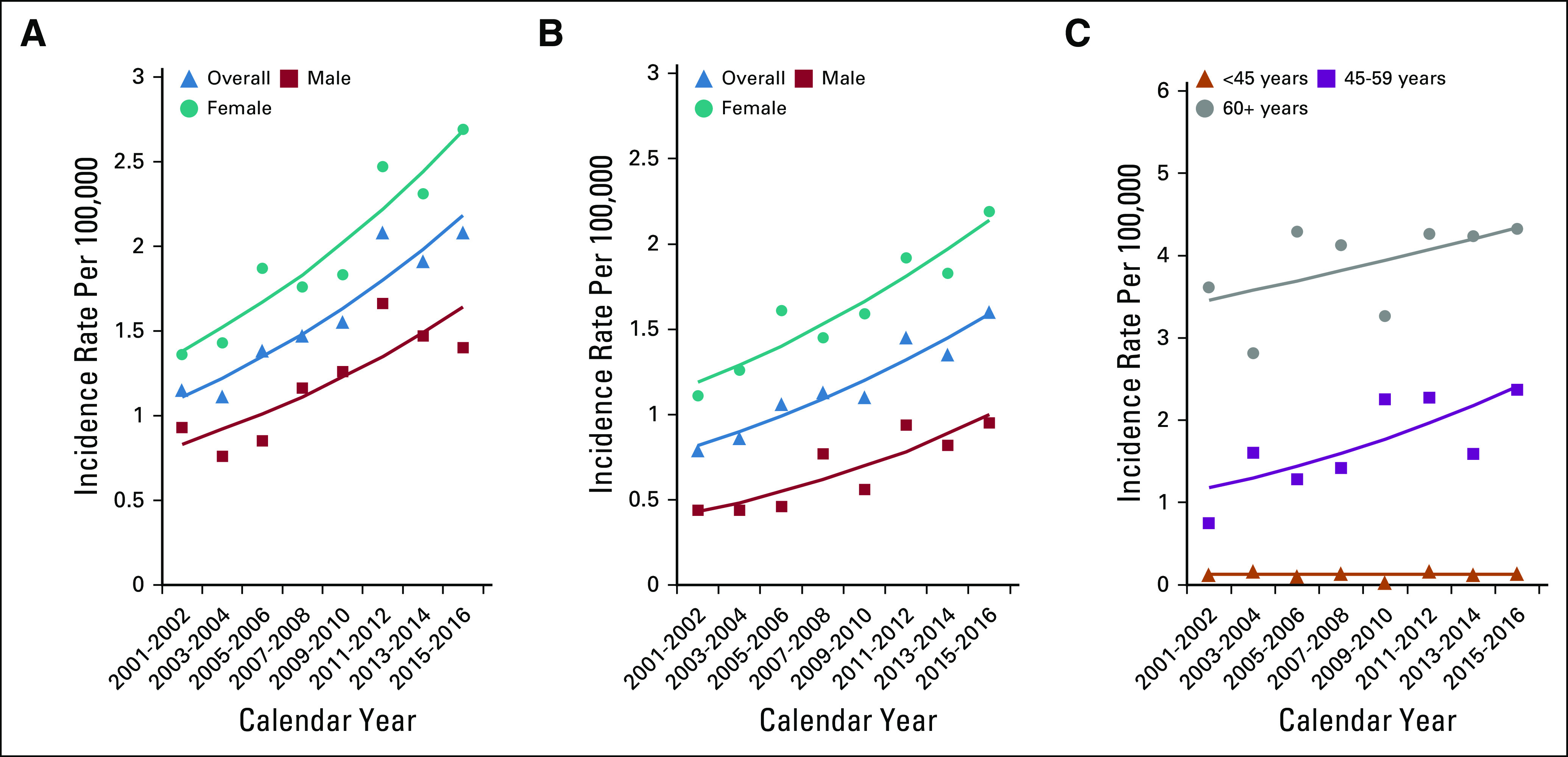
(A) Anal cancer in the overall general population and by sex, (B) SCCA in the overall general population and by sex, (C) SCCA among the overall general population and by age group.

**FIG 2:**
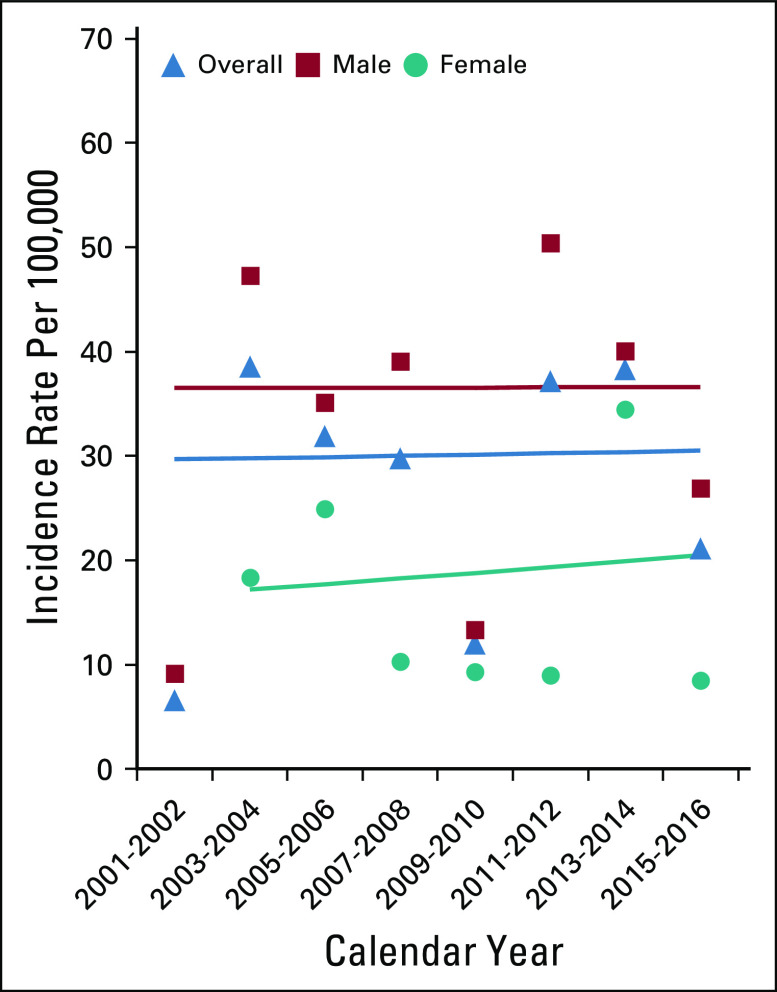
SCCA among PLWH overall and by sex.

Trends in incidence rates of anal cancer and SCCA among the general population are presented by sex and age (Fig 1). Between 2001 and 2016, among the general population, a rapid rise in anal cancer (APC = 4.9 and *P* = .0003) and SCCA (APC = 4.9 and *P* = .0002) incidence occurred (Fig 1A). In stratified analyses among the general population, SCCA incidence increased rapidly among men (APC = 6.2 and *P* = .0052) and women (APC = 4.3 and *P* = .0005) (Fig 1B). In age-stratified analysis, the rise was marked among individuals age 45-59 years (APC = 5.3 and *P* = .0425) (Fig 1C). Although incidence rates of anal cancer and SCCA are higher among PLWH, trends in incidence rates for anal cancer (APC = 1.8 and *P* = .6067) and SCCA remained stable (APC = 0.2 and *P* = .9602) in this group during the study period (Fig 2).

The mortality rate for anal cancer in the general population was 0.10 per 100,000 (Table 4). Among women, the anal cancer mortality rate was 0.14 per 100,000 compared with 0.06 per 100,000 in men. The mortality rate increased from 2000-2005 (0.09 per 100,000) to 2012-2016 (0.15 per 100,000). An increase in anal cancer mortality was observed for the general population (APC = 3.9 and *P* = .27), although this increase was not statistically significant (Table 4).

**TABLE 4:**
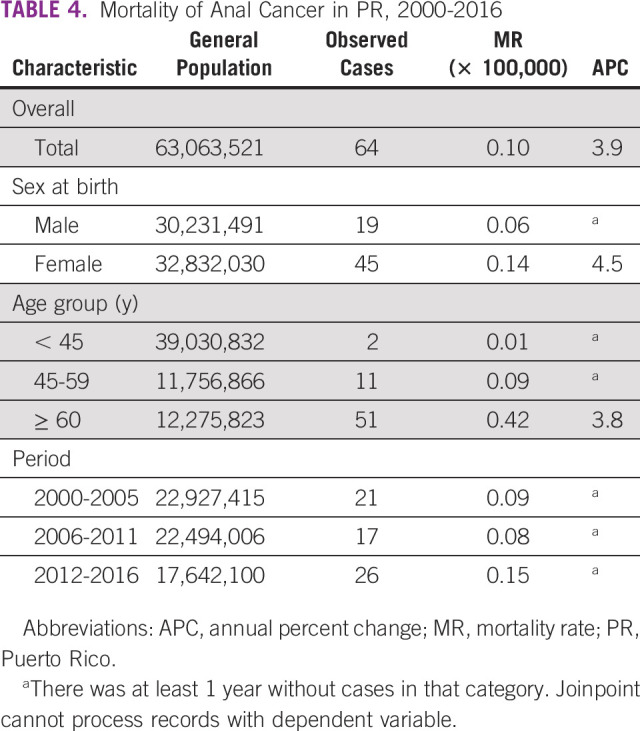
Mortality of Anal Cancer in PR, 2000-2016

Abbreviations: APC, annual percent change; MR, mortality rate; PR, Puerto Rico.

^a^ There was at least 1 year without cases in that category. Joinpoint cannot process records with dependent variable.

Five-year relative survival of SCCA among PLWH was 56.9%, whereas, among the general population, it was 66.8%. In PLWH, 5-year relative survival was 55.1% for men and 67.9% for women. Individuals diagnosed with SCCA in the earlier study period (2000-2007) had a 61.7% relative survival versus 71.1% of people diagnosed during 2008-2012. PLWH with a regional/distant stage of cancer diagnosis had a relative survival of 52.0%, whereas individuals diagnosed with localized disease had a 61.2% relative survival. When analyzed by age groups, 5-year relative survival was lower for individuals age < 45 years (51.0%). Similar results were observed in the general population and for the other time intervals at 1-year and 3-year relative survival (Table 5).

**TABLE 5:**
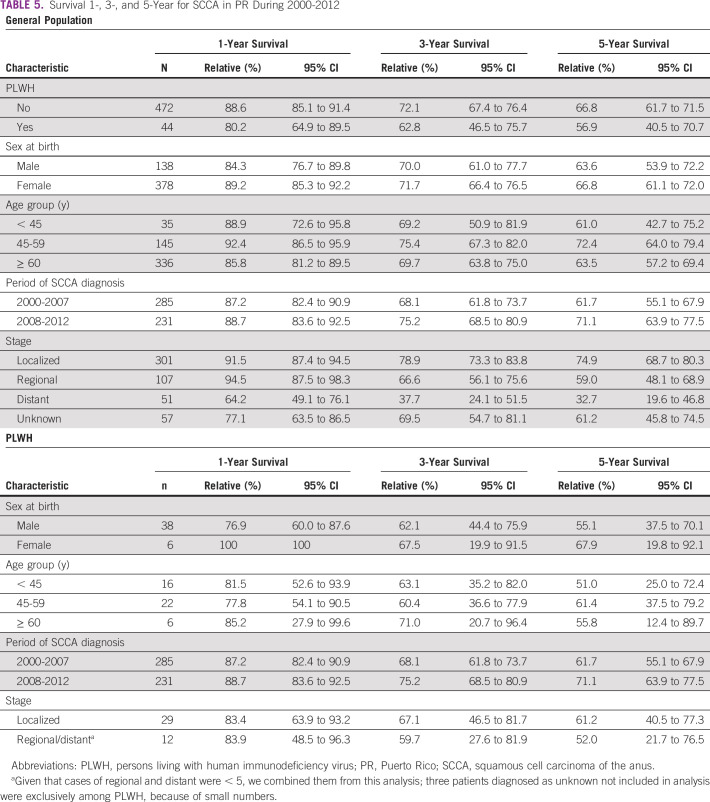
Survival 1-, 3-, and 5-Year for SCCA in PR During 2000-2012

Abbreviations: PLWH, persons living with human immunodeficiency virus; PR, Puerto Rico; SCCA, squamous cell carcinoma of the anus.

^a^ Given that cases of regional and distant were < 5, we combined them from this analysis; three patients diagnosed as unknown not included in analysis were exclusively among PLWH, because of small numbers.

Regarding the excess risk of death in multivariate analyses, no significant differences were observed by HIV infection status or by age group in men or women. Marginally significant differences (.05 > *P* < .10) were seen in the period of SCCA diagnosis in both men and women, where those diagnosed in the most recent period (2008-2012) had a lower risk of death than their counterparts diagnosed in the initial study period (2000-2007) (RER_men_, 0.48, 95% CI, 0.23 to 0.01; RER_women_, 0.68, 95% CI, 0.45 to 1.03). The largest differences in SCCA risk of death were seen by stage at diagnosis, where men diagnosed at a distant stage had almost seven times the risk of death (RER_men_, 7.57; 95% CI, 2.36 to 24.25) compared with those diagnosed at a localized stage. Similar results were observed among women, whereas those diagnosed with a regional (RER_women_, 2.1; 95% CI, 1.28 to 3.45) and distant stage (RER_women_, 5.15; 95% CI, 3.08 to 8.60) had a higher risk of death than those who were diagnosed in a localized stage (Table 6).

**TABLE 6:**
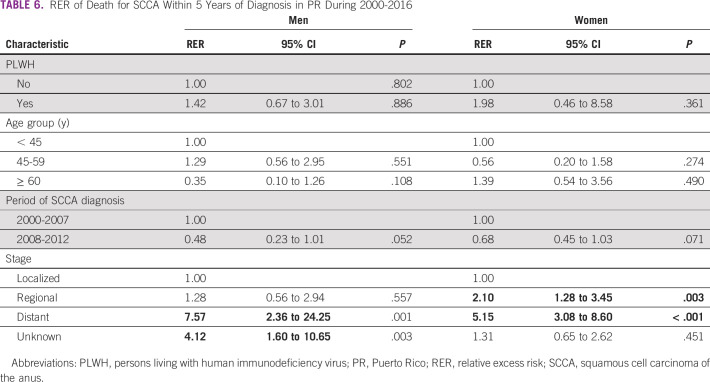
RER of Death for SCCA Within 5 Years of Diagnosis in PR During 2000-2016

Abbreviations: PLWH, persons living with human immunodeficiency virus; PR, Puerto Rico; RER, relative excess risk; SCCA, squamous cell carcinoma of the anus.

## DISCUSSION

To the best of our knowledge, our study is the first to comprehensively assess incidence, mortality, and survival of anal cancer among PLWH and the general population living in PR. Our findings indicate that the rates of SCCA have increased dramatically among the general population. Although the incidence rate remains substantially greater among PLWH, its rise seems to have moderated in this risk group. The high prevalence of HIV in PR and rapid rise in incidence imply that implementation of evidence-based anal cancer screening is needed to curb the rising disease burden.

SCCA risk among PLWH in PR is substantially elevated compared with the general population. However, our results showed that SCCA rates are not increasing among PLWH, but marked increase has occurred among the general population. Our findings show that the incidence of SCCA is increasing more rapidly in PR (population mostly of Hispanic origin) than in the United States.^5^ Furthermore, although 85%-90% of anal cancer cases are SCCA,^1^ in PR, our results show that this proportion was surprisingly lower than expected (73%). This increase in SCCA incidence in the general population could be due to an increase in SCCA-related risk factors, including high-risk HPV infection, anal receptive sex, smoking, and increased number of sexual partners.^22,23^

Regarding the 5-year relative survival rate of SCCA, our study showed a 5-year survival of 66.8% for the general population and 56.9% for PLWH. Although caution is recommended with these comparisons, our estimates are consistent with the national estimates of 68.3% among the general population of the United States.^24^ It has been shown that SCCA has better survival than other anal cancer histologies, which could affect the comparison between SCCA and anal cancer survival.^3,4^

Meanwhile, those diagnosed in a regional and distant stage had a higher risk of death than those who were diagnosed in a localized stage. The fact that SCCA survival in this population was influenced by the stage of diagnosis, rather than HIV status, highlights the importance of prevention and early detection since an anal cancer diagnosis is often delayed as a result of confounding symptoms with other rectal conditions,^25^ which demonstrates the necessity to prevent this malignancy through screening and HPV vaccination. Nonetheless, this is concerning as currently no national evidence-based screening guidelines are available for anal cancer screening. Some expert groups recommend anal cytology as a primary screening tool for at-risk groups, such as PLWH, in settings where high-resolution anoscopy (HRA) followed by the treatment of anal high-grade squamous intraepithelial lesions (HSILs) is available;^26,27^ however, the harms versus benefits of screening these individuals and optimal screening algorithms still remain unclear.^27–29^ National studies such as the ANCHOR trial (ClinicalTrials.gov identifier: NCT02135419) are currently evaluating the efficacy of treating anal HSIL for SCCA prevention. One of the biggest obstacles for the implantation of an anal cancer screening program is that the HRA is difficult to perform and involves additional training for the physicians. Currently, the number of clinicians with adequate experience for diagnosing and managing anal precancerous lesions remains inadequate. Educational efforts to train physicians should be a priority for implementing anal cancer screening programs.^28,29^

Another factor that could affect survival among our population could be that a high percentage of the Puerto Rican population (with an even higher proportion of PLWH) has public health coverage.^30,31^ This could potentially lead to more advanced stage at diagnosis and/or delayed access to preventive and treatment services as research has shown that patients with public health coverage in PR experience longer waiting times to schedule an appointment, to see a specialist, and to receive cancer treatment.^10,30,32–35^ More studies are needed to understand how type of health coverage affects survival in PR.

Given that around 90% of SCCA cases are due to an infection with high-risk HPV,^3^ this cancer could be prevented through HPV vaccination. The Centers for Disease Control and Prevention recommends routine HPV vaccination for girls and boys age 11-12 years, with catch-up vaccination until age 26 (including PLWH), with most recent recommendations including persons until the age 45 years.^36^ The HPV vaccines prevent infections with high-risk HPV types such as 16 and 18, which have been found to cause around 70% of cervical HPV-related cancers.^37^ In PR, data from the 2016 National Immunization Survey-Teen show that around 52.8% of teenagers age 13-17 years were up-to-date with HPV vaccination coverage, as compared to 49.9% in the United States.^38^ Nonetheless, a recent HPV vaccine school entry policy established in 2018 in PR should have an impact on increasing uptake among adolescents within the next years, as the availability of school mandates seems to be a stronger driver for the improvement in vaccination rates.^39^

Future studies evaluating other factors that may affect future anal cancer trends must be considered. Cigarette smoking, increased body mass index, and prior lower genital tract dysplasia or malignancy among females have been identified as factors that could contribute to the increased incidence of SCCA rates.^22,40–43^ Increased smoking cessation campaigns and screening for high-risk populations may have an impact on reducing SCCA rates.

Limitations for our study include that we could not include individual-level data such as HPV infection, smoking status, ART use, and other HIV disease markers (CD4 count and HIV viral load), and so, we can only hypothesize on how these and other covariates may affect the incidence and survival of anal cancer in our population. Despite this, our study has several strengths. Considering we used information from the PRCCR and the HIV/AIDS Surveillance System, we have population-based data from PR, which permits us to estimate incidence, mortality, and survival at the population level and for PLWH and to describe high-risk groups, such as MSM, in our analysis.

In conclusion, our study findings show that in PR, SCCA incidence and mortality rates are rising in the general population. Future studies are needed to understand etiological reasons for these trends. Meanwhile, increased risk of SCCA and lower 5-year relative survival among PLWH as compared to the general population highlight an important cancer health disparity in this group. The fact that the RER of death for SCCA 5 years postdiagnosis was affected by stage at diagnosis but not by HIV status, evidences the importance of assessing strategies to facilitate early diagnosis of SCCA, in both PLWH and the general population. Our study results imply the need to determine the optimal anal cancer screening strategies in PLWH in PR, whereas continuous improvement in HPV vaccine uptake is also needed, to have an impact on SCCA trends over the next decades.
